# From defects to catalysis: mechanism and optimization of NO electroreduction synthesis of NH_3_

**DOI:** 10.3389/fchem.2024.1452689

**Published:** 2024-08-30

**Authors:** Gan Linling, Zhen Liao, Huimei Zhang, Jinxia Jiang, Zhikai Chen

**Affiliations:** ^1^ Chongqing Medical and Pharmaceutical College, Chongqing, China; ^2^ School of Chemistry and Chemical Engineering, Chongqing University, Chongqing, China

**Keywords:** defect engineering, nitric oxide reduction reaction, vacancy defects, doping defects, electrocatalysis

## Abstract

Ammonia (NH₃) is a crucial industrial raw material, but the traditional Haber-Bosch process is energy-intensive and highly polluting. Electrochemical methods for synthesizing ammonia using nitric oxide (NO) as a precursor offer the advantages of operating under ambient conditions and achieving both NO reduction and resource utilization. Defect engineering enhances electrocatalytic performance by modulating electronic structures and coordination environments. In this brief review, the catalytic reaction mechanism of electrocatalytic NO reduction to NH_3_ is elucidated, with a focus on synthesis strategies involving vacancy defects and doping defects. From this perspective, the latest advances in various catalytic reduction systems for nitric oxide reduction reaction (NORR) are summarized and synthesized. Finally, the research prospects for NO reduction to NH₃ are discussed.

## 1 Introduction

Ammonia, a colorless and strongly irritating chemical substance, not only plays a crucial role in the chemical industry, but also serves as a highly anticipated carbon neutral energy carrier (Li et al., 2020a; Zhao et al., 2023). In numerous industrial production processes, ammonia plays an irreplaceable role, from the manufacturing of fertilizers to the production of chemical raw materials, and then to the preparation of coolants and refrigerants, ammonia plays a core role (Guo et al., 2021; Cheon et al., 2022). More noteworthy is that ammonia also occupies an extremely important position in human daily life. From food preservation to pharmaceutical production, and even to the development of clean energy, ammonia provides strong support for the progress and development of human society with its unique properties and functions. However, despite the undeniable importance of ammonia, its production currently faces enormous challenges (Yang et al., 2023a; Chen et al., 2024a; Kim et al., 2024). At present, the production of ammonia worldwide mainly relies on the Haber Bosch process, which is a reaction carried out under high temperature and pressure (Lin et al., 2022; Chen et al., 2023a). It relies on a large amount of fossil energy as the driving force and also generates a large amount of carbon dioxide emissions. This not only puts enormous pressure on global climate change, but also exacerbates the energy crisis, making the production cost of ammonia high and difficult to achieve large-scale promotion and application (Chen et al., 2024b; Inta et al., 2024). Therefore, seeking more environmentally friendly, efficient, and economical ammonia production methods has become an important issue in current scientific research and industrial fields (Xiong et al., 2022; Kong et al., 2024). This not only requires scientists to conduct in-depth research and exploration, but also requires the joint efforts of the government, enterprises, and society to promote the innovation and development of ammonia production technology, in order to achieve green, low-carbon, and efficient production of ammonia, and contribute more to the sustainable development of humanity (Badalyan et al., 2023; Chen et al., 2023b).

**FIGURE 1:**
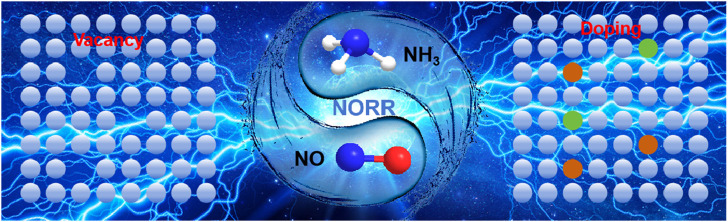
Electrocatalytic NORR material design: vacancy defects and doping defects.

In recent years, with the increasing global pursuit of sustainable energy and environmental protection technologies, electrocatalytic conversion of N_2_ to NH_3_ (NRR) has attracted widespread attention from the scientific research community as a green and energy-saving method for synthesizing ammonia (Guo et al., 2024). This technology is expected to not only solve the problems of high energy consumption and pollution in traditional ammonia synthesis processes, but also provide stable and reliable ammonia sources for agriculture, chemical industry and other fields. However, despite the enormous potential of electrocatalytic NRR, it faces many challenges in practical applications (Mangini et al., 2024). Firstly, the nitrogen triple bonds in nitrogen (N2) molecules have extremely high dissociation energy (941 kJ mol^−1^), which means that a large amount of energy is required in the electrocatalytic process to activate them and convert them into easily reactive intermediate states (Wang et al., 2024). Secondly, the solubility of nitrogen in water is extremely low, which limits the contact area between nitrogen and catalyst in electrocatalytic reactions, further reducing the efficiency of NRR (Yan et al., 2023; Dai et al., 2024). Finally, in aqueous solutions, electrocatalytic reactions are often accompanied by intense competitive hydrogen evolution reactions (HER), which can lead to a decrease in current efficiency and catalyst deactivation (Xie et al., 2023; Sun et al., 2024). Researchers have been working hard to find solutions to these problems. One possible approach is to use NO (nitric oxide) as a raw material for electrochemical reduction. Compared with N_2_, NO has stronger reactivity, therefore, thermodynamically, NORR is more feasible than NRR electro synthesis of NH_3_ (Li et al., 2023a). Meanwhile, as an atmospheric pollutant, NO’s environmental pollution problem is becoming increasingly prominent. NO has high chemical activity and is prone to react with oxygen in the air to generate nitrogen dioxide, which in turn forms photochemical smog and causes serious damage to the atmospheric environment (Wang et al., 2023a). In addition, NO reacts with water molecules in the atmosphere to generate nitric acid and nitrite, which enter the surface through dry and wet deposition, leading to soil and water acidification and long-term negative impacts on the ecological environment (Wang et al., 2023b). This approach not only avoids the difficulties in the NRR process, but also effectively utilizes NO, a pollutant, to achieve the resource utilization of waste. Therefore, catalyst design strategies with excellent synthetic performance have attracted great attention from researchers (Long et al., 2020). Among the many considerations in catalyst design, defects play a particularly significant role, although their inherent complexity poses significant challenges for related research (Li et al., 2024; Liang et al., 2024). Specifically, the type, quantity, and spatial distribution of defects have a profound impact on catalytic performance, which is regulated by various factors such as the basic composition of the material, the choice of synthesis technology, and the setting of reaction conditions. It is worth noting that defects are particularly common in heterogeneous/amorphous nanomaterials. They regulate the electronic structure and coordination environment of the electrocatalyst, thereby affecting the adsorption energy of reactants on its surface, which is expected to optimize and improve the electrocatalytic performance (Li et al., 2022a). Vacancy defects and doping defects have attracted widespread attention in the design of NORR catalytic materials, as shown in Figure 1.

In this concise review, we briefly outline the reduction mechanism of NO to NH_3_, and then discuss the preparation and characterization of defective materials (vacancy defects and doping defects). In addition, we conducted in-depth research on the effects of vacancy defects and doping defects on the NORR process, elucidating their structure-activity relationships. Finally, we emphasize the prospects and challenges of vacancy defects and doping defects, covering controllable synthesis, *in situ* characterization, and practical applications.

## 2 The reaction principle and mechanism of electrocatalytic reduction of NO to ammonia

The process of electrocatalytic NO reduction to NH_3_ is a highly complex and intricate electrochemical transformation pathway involving multiple consecutive and interrelated steps, which collectively determine the overall reaction efficiency and selectivity (as shown in Equations 1–4) (Ren et al., 2022; Zhao et al., 2022; Chen et al., 2023c). This process not only requires effective adsorption of NO molecules on the electrode surface but also necessitates multi-step electron transfer and proton transport to progressively achieve NO reduction. The reaction initiates with the physical or chemical adsorption of NO gas molecules onto the electrocatalyst surface, forming a stable adsorbed NO state. This initial step is critical for the subsequent reactions as it determines the extent of NO molecules’ interaction with the catalyst surface and their reactivity. Once NO molecules are successfully adsorbed on the catalyst surface, they undergo a stepwise reduction process. Firstly, the adsorbed NO molecules capture one or more electrons to form the intermediate product NOH. This step is one of the key electron transfer stages and depends on the electronic structure of the catalyst surface and the reaction conditions. The capture of electrons alters the chemical nature of the NO molecules, making further reduction possible. Subsequently, the NOH intermediate continues to accept electrons and protons, gradually converting into NH_2_O. This step also requires the support of specific adsorption sites and the electronic structure of the catalyst surface to ensure effective electron and proton transfer. The formation of NH₂O marks the critical phase of the NO reduction process, laying the foundation for the final production of NH₃ and water (H_2_O). Ultimately, NH_2_O undergoes successive electron and proton transfer steps, progressively reducing to NH₃ and H_2_O. This final step concludes the entire reaction process and represents the ultimate goal of electrocatalytic NO reduction to ammonia. Through the optimization of reaction conditions and catalyst design, the efficiency and selectivity of this step can be further enhanced, thereby achieving more efficient and greener ammonia production (Ren et al., 2022).

$$\text{NO}\left(g\right)\to \text{NO}\left(\text{ads}\right)$$ (1)

$$\text{NO}\left(\text{ads}\right)+e^{‐}\to \text{NOH}\left(\text{ads}\right)$$ (2)

$$\text{NOH}\left(\text{ads}\right)+e^{‐}+H+\to NH_{2}O\left(\text{ads}\right)$$ (3)

$$NH_{2}O\left(\text{ads}\right)+3e^{‐}+3H+\to NH_{3}+H_{2}O$$ (4)

In this process, the precise selection of catalysts is crucial for achieving high reaction rates and selectivity. Widely studied catalysts include various metal electrodes (such as platinum, ruthenium, copper, etc.) and nanomaterials, particularly metal nanoparticles supported on carbon materials like graphene (Zhang Y. et al., 2024c). The performance of these catalysts is primarily influenced by their surface adsorption sites and electronic structures, which directly determine the adsorption capacity of NO molecules and the efficiency of electron transfer from the electrode to the NO molecules (Mou et al., 2021). The exploration of defect engineering provides a new perspective for enhancing the reaction efficiency and selectivity of these materials. Defect materials, especially catalysts with specific defect structures, significantly impact electrocatalytic reactions by altering the electronic structure of the material, increasing surface active sites, and promoting charge and mass transfer.

## 3 Application of defective materials in electrocatalytic NO reduction: vacancy defects and doping defects

In the in-depth study of materials science, the concept of defects has gradually shifted from being perceived as “imperfections” to “potential opportunities” (Zheng et al., 2022). Defects, which are localized irregularities or incompleteness in the atomic, ionic, or electronic structures of materials, have now become critical breakthroughs for exploring new material properties and optimizing material performance (Li W. et al., 2020; Li et al., 2022b; Yang et al., 2023c). In the field of NORR, defect materials, with their unique physical and chemical properties, exhibit tremendous potential and application value. Defects typically refer to structural imperfections or non-ideal states in materials, including vacancies, impurities, lattice distortions, and other forms, which can significantly influence the electronic structure, surface properties, and catalytic activity of materials. In the electrocatalytic NO reduction reaction, defect structures can enhance the adsorption capacity of NO molecules by providing additional active sites, thus facilitating the activation of NO (Sun et al., 2023). For example, introducing oxygen vacancies in TiO₂ can significantly improve the adsorption and activation efficiency of NO. Moreover, defect materials can optimize electron transfer pathways, alter the electronic structure of the material, and reduce the reaction overpotential. Additionally, defect structures can optimize proton transfer and stabilize intermediate products, thereby increasing the reaction efficiency (Chen et al., 2023d). Finally, defect materials possess high structural stability, maintaining high catalytic activity over prolonged reactions, enhancing corrosion and oxidation resistance, and extending the catalyst’s lifespan (Chen et al., 2023e). By employing sophisticated characterization techniques such as Scanning Electron Microscopy (SEM), Transmission Electron Microscopy (TEM), X-ray Diffraction (XRD), Electron Paramagnetic Resonance (EPR), researchers are able to delve deep into the detailed microstructures and chemical compositions of defective materials. These advanced characterization methods not only provide crucial information about the types, distribution, and formation mechanisms of defects, but also assist scientists in establishing the structure-performance relationships between defects and the electrocatalytic properties of materials. Specifically, SEM and TEM offer high-resolution morphology and structural images, enabling researchers to observe defect distribution and morphology at the nanoscale; XRD is utilized to determine the crystal structure and defect states of materials; EPR detects electronic defects and unpaired electrons in materials, thereby providing information about the electronic environment of defects. By integrating these characterization techniques, researchers can systematically analyze and comprehend the role of defect engineering in electrocatalytic processes, thus guiding the design and optimization of new efficient catalysts (Yan et al., 2022).

**FIGURE 2:**
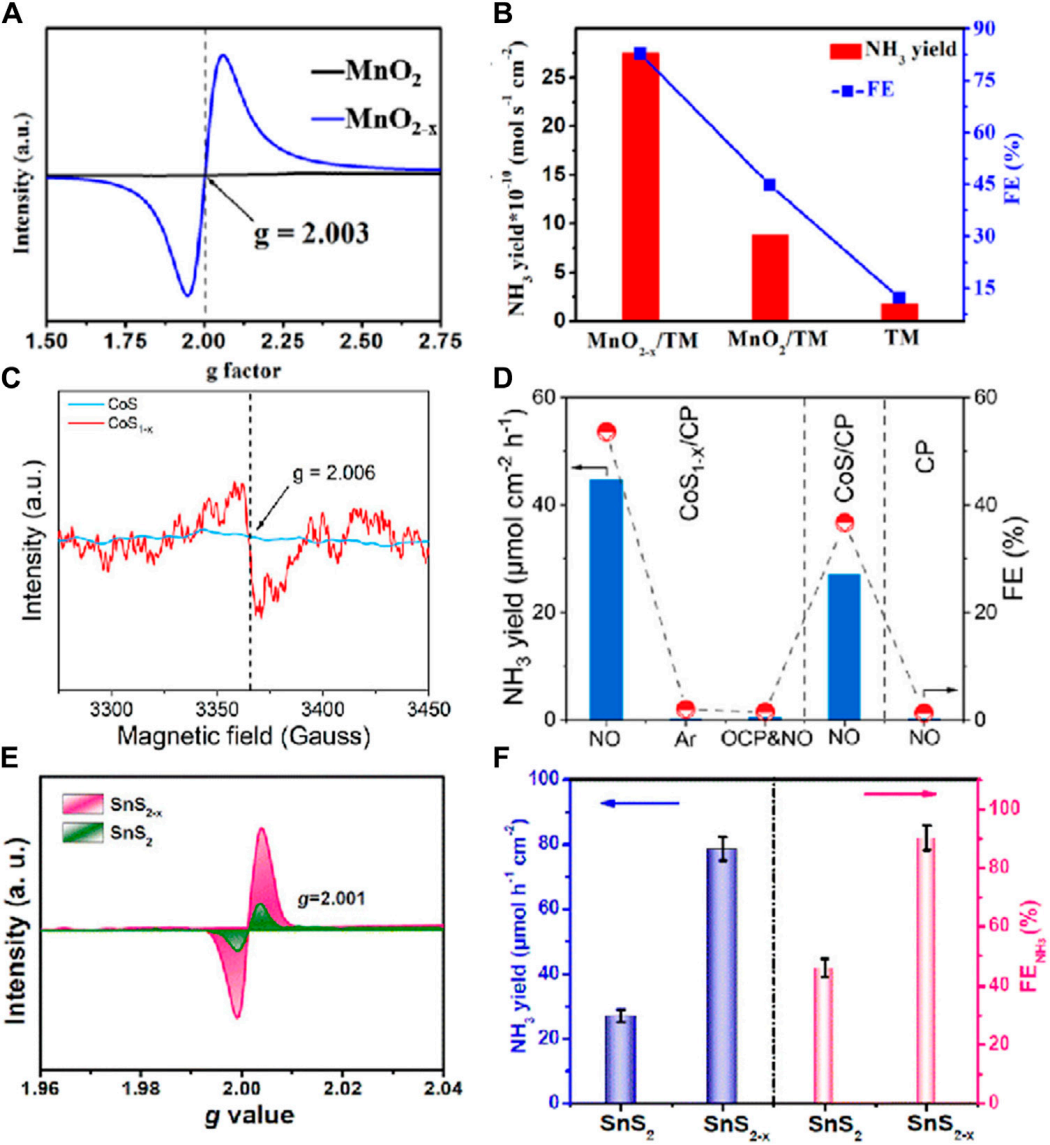
**(A)** EPR spectra of MnO_2_ and MnO_2-x_; **(B)** NH_3_ yields and FEs with different electrodes at −0.7 V for 1 h bulk electrolysis (Li et al., 2022b); **(C)** EPR spectra of CoS and CoS_1-x_; **(D)** Comparison of NH_3_ yield rates and FEs with different electrodes under different conditions (Zhang et al., 2022); **(E)** EPR spectra of SnS_2_ and SnS_2-x_; **(F)** Comparison of the NORR performance between SnS_2_/CC and SnS_2-x_/CC at −0.7 V (Li et al., 2023b).

In the field of electrocatalysis, the introduction of defects provides new insights for the design of electrocatalysts. A vacancy defect refers to a position in a crystal structure where one or more atoms are missing. Specifically, when an atom is removed from its normal lattice site, a vacancy defect is formed. This is one of the most common types of defects in materials and can significantly alter the electronic structure and surface properties of the material, thereby affecting the activity and selectivity of electrocatalytic reactions (Han et al., 2023; Xiao et al., 2023). For instance, in the electrocatalytic reduction of NO to NH₃, the rational regulation of vacancy defects in materials can optimize the adsorption and activation capabilities of the catalyst for NO molecules, enhancing reaction efficiency and product purity. Additionally, vacancy defects can lower the activation energy of the reaction, allowing the electrocatalytic process to proceed at lower overpotentials, thus reducing energy consumption and improving energy utilization efficiency. For example, Li et al. (2022c) synthesized MnO₂-x nanowire arrays with oxygen vacancies (VO) on titanium mesh (TM), and detected the VO on the MnO₂ surface using electron paramagnetic resonance (EPR) spectroscopy. In Figure 2A, the signal of MnO_2-x_ is attributed to paramagnetic VO, with a g-peak value of 2.003 corresponding to VO in its lattice. EPR results confirmed the presence of more surface active oxygen species and VO in MnO_2-x_. In 0.2 M Na₂SO₄, this catalyst achieved an NH₃ yield of up to 27.51 × 10⁻^1^⁰ mol s⁻^1^ cm⁻^2^, more than double the increase (vs. 8.83 × 10⁻^1^⁰ mol s⁻^1^ cm⁻^2^), with a Faradaic efficiency of up to 82.8%, nearly double the previous value (vs. 44.8%) (Figure 2B). These results highlight the critical role of VO in enhancing the NORR catalytic activity of MnO_2_ (Li et al., 2022b). Zhang et al. (2022) synthesized CoS1-x with sulfur vacancies using a combination of hydrothermal and plasma treatments. To confirm the presence of sulfur vacancies, EPR tests were conducted, showing a significant EPR signal at g = 2.006, indicating the presence of sulfur vacancies in the material (Figure 2C). In a 0.2 M Na_2_SO_4_ electrolyte (Figure 2D), this CoS1-x exhibited a higher NH_3_ yield (44.67 μmol cm⁻^2^ h⁻^1^) and higher Faradaic efficiency (53.62%) at −0.4 V compared to CoS (27.02 μmol cm⁻^2^ h⁻^1^; 36.68%) (Zhang et al., 2022). Li et al. employed defect engineering strategies to develop an SnS₂-x catalyst rich in S-vacancy (VS) defects. EPR spectroscopy (Figure 2E) revealed that SnS₂-x displayed a stronger g-signal (2.001) than SnS₂, further confirming the abundance of VS in SnS₂-x. The NORR performance of the materials was evaluated under the same electrolysis conditions (Figure 2F). The NH₃ yield of SnS_2-x_/CC (78.6 μmol h⁻^1^) was 2.8 times that of SnS₂/CC, and the FE (90.3%) was 1.8 times that of SnS_2_/CC, with sulfur vacancies significantly enhancing the NORR performance of SnS₂-x. In summary, vacancy defects demonstrate significant advantages in the NORR (Li et al., 2023b). These defects not only enhance catalytic activity by modulating the electronic structure of the catalyst but also lower reaction barriers, thereby promoting the activation and conversion efficiency of NO molecules. Furthermore, vacancy defects enhance the interaction between the catalyst and reactants, improving the stability and durability of the catalyst (Mushtaq et al., 2024).

**FIGURE 3:**
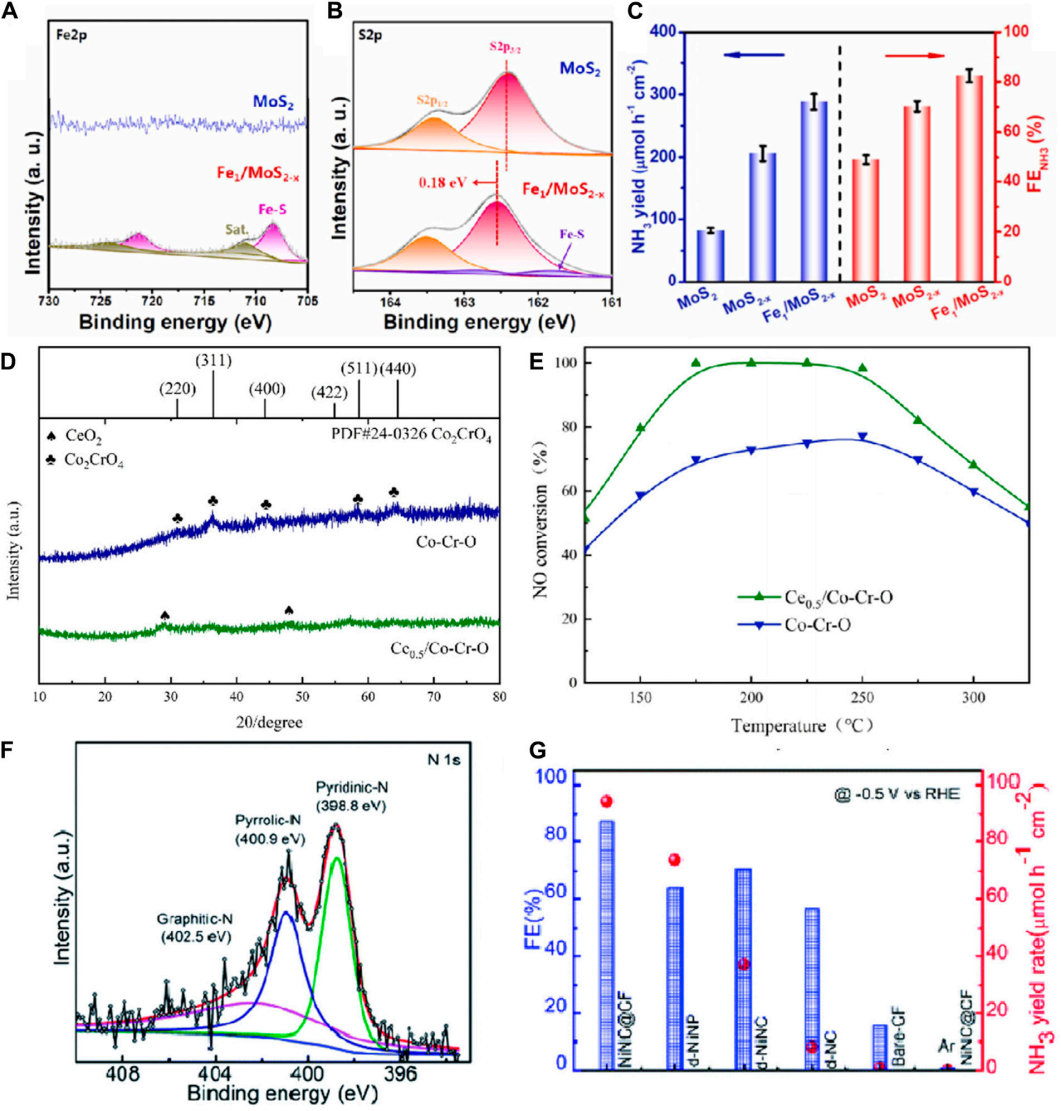
**(A,B)** XPS spectra of MoS2 and Fe1/MoS2-x: **(A)** Fe2p, **(B)** S2p.; **(C)** NORR performances of MoS2, MoS2-x, and Fe1/MoS2-x (Chen et al., 2023f); **(D)** XRD of Co-Cr-O catalyst and Ce0.5/Co-Cr-O catalyst; **(E)** NO-conversion of Co-Cr-O catalyst and Ce0.5/Co-Cr-O catalyst (Jin et al., 2023); **(F)** High resolution XPS spectra of N 1s; **(G)** Comparison of FE and NH_3_ yields on various electrodes after 1 h of NORR (Muthusamy et al., 2022).

Doping defects exhibit significant potential in the NORR. A doping defect refers to the introduction of specific impurity atoms or ions into a crystal structure. These impurity atoms or ions either replace some of the host atoms in the lattice or occupy interstitial sites in the crystal. By introducing heteroatoms such as nitrogen (N), sulfur (S), and phosphorus (P) into catalytic materials, doping defects can precisely regulate the electronic structure and surface chemical properties of the materials, thereby significantly enhancing catalytic activity and reaction efficiency (He et al., 2022; Chen et al., 2023f; Zhang et al., 2024a). For instance, nitrogen-doped graphene and carbon nanotubes, due to their unique electronic properties and high active sites, markedly enhance the adsorption and activation capabilities of NO molecules. Specifically, pyridinic nitrogen and graphitic nitrogen sites in these materials demonstrate high activity during the NO reduction process. Sulfur doping, by incorporating sulfur atoms, increases the conductivity and reactive sites of the material, optimizes electron transfer pathways, and improves reaction efficiency. Similarly, phosphorus-doped carbon materials exhibit excellent catalytic performance; the introduction of phosphorus atoms alters the electronic structure of the material, enhancing the adsorption capacity and catalytic activity for NO. Metal-doped carbon materials, such as those doped with iron (Fe), cobalt (Co), and nickel (Ni), form metal-nitrogen-carbon (M-N-C) structures, significantly boosting catalyst activity. Among these, Fe-N-C catalysts with Fe-N_x_ sites show exceptionally high catalytic performance in the NO reduction process. These doped defect materials not only enrich the adsorption and activation sites for NO but also optimize electron transfer pathways and reduce reaction overpotentials by altering the electronic structure and conductivity of the materials, thereby significantly enhancing the efficiency of NO reduction to NH₃ (Guo et al., 2021; Yang et al., 2023b; Zhang et al., 2024b). Chen et al. (2023f) prepared Fe-doped MoS₂ through a simple one-step hydrothermal method. The XPS-Fe2p spectrum of Fe1/MoS₂-x in Figures 3A, B showed distinct peaks corresponding to Fe-S bonds, which were absent in the original MoS₂. Additionally, the S2p spectrum exhibited characteristic double peaks of Fe-S bonds, confirming the successful doping of Fe. Figure 3C illustrates the performance of Fe1/MoS₂-x as an efficient NORR catalyst, showing a maximum NH₃ Faradaic efficiency of 82.5% and an NH₃ yield of 288.2 μmol h⁻^1^ cm⁻^2^ at −0.6 V vs RHE. The doping of Fe played a crucial role in modulating the electronic structure of MoS₂, thereby enhancing its conductivity (Chen et al., 2023f). Jin et al. (2023) prepared a cerium-doped cobalt-chromium oxide catalyst (Ce₀.₅/Co−Cr−O) using a co-precipitation method. As shown in Figure 3D, the diffraction peaks at 2θ = 28.5° and 47.5° correspond to the (111) and (220) planes of CeO₂ (PDF#81–0,792), with no evident Co₂CrO₄ on the surface, indicating the successful doping of Ce and its alteration of the catalyst’s crystal structure. The Ce₀.₅/Co−Cr−O catalyst exhibited optimal NO conversion rates, exceeding 90% from 175°C to 250°C (Figure 3E). Across the entire temperature range, the NO conversion rate of the Ce₀.₅/Co−Cr−O catalyst surpassed that of the Co−Cr−O catalyst. This finding indicates that Ce doping effectively enhances NO conversion rates. Doping with Ce significantly altered the catalyst’s surface morphology, reduced crystallinity, and increased specific surface area. A larger specific surface area facilitates the high dispersion of acidic sites, promoting NH₃ adsorption and desorption, thereby enhancing its NORR performance (Jin et al., 2023). Muthusamy et al. (2022) synthesized MOF-derived zero-valent nickel nanoparticles encapsulated with nitrogen-doped carbon nanostructures on carbon fibers through a stepwise process. In the XPS-N 1s spectrum (Figure 3F), three peaks were observed at 398.8, 400.9, and 402.5 eV, corresponding to pyridinic nitrogen, pyrrolic nitrogen, and graphitic nitrogen, respectively. Among all electrodes, NiNC@CF demonstrated relatively superior NORR activity (Figure 3G), with an NH₃ yield of 94 μmol h⁻^1^ cm⁻^2^ and a significantly high Faradaic efficiency (87%) at −0.5 V vs. RHE. This enhancement is attributed to the unique core-shell arrangement in the NiNC catalyst, which exhibits synergistic effects between metallic Ni and N-doped nanostructures, improving electrocatalytic activity and chemical stability (Muthusamy et al., 2022).

Defect materials hold great promise in the electrocatalytic reduction of NO. By precisely controlling the type, concentration, and distribution of defects, the catalytic performance of materials can be further optimized. Combined with other optimization strategies, such as nanostructure design and the development of composite materials, more efficient NO reduction to ammonia can be achieved. As the mechanistic study of defect materials deepens, more efficient and stable catalysts will be developed, advancing the practical application of electrocatalytic NO reduction to ammonia technology.

## 4 Conclusion and outlook

In the conversion of NO to NH_3_, the introduction of vacancy defects and doping defect materials is regarded as a crucial strategy for enhancing catalytic performance and reaction efficiency, showing significant research and application prospects. Vacancy defects, including cationic and anionic vacancies, can effectively enhance the adsorption capacity of NO molecules and optimize charge transfer and catalytic efficiency by modulating the electronic structure of the catalyst. This accelerates the conversion process from NO to NH_3_. Through precise control of the type, distribution, and density of vacancy defects, engineered catalysts can significantly improve their catalytic activity and selectivity. Doping defects, which involve the addition of specific amounts of impurity atoms or ions, can not only adjust the electronic structure and lattice parameters of the materials but also increase the stability of vacancies and reduce the energy required for vacancy formation, thereby optimizing the catalytic performance during the NO reduction process. The implementation of doping strategies allows catalysts to maintain high catalytic activity and stability under harsh reaction conditions such as high temperatures and pressures. However, the application of vacancy and doping defect materials in the conversion of NO to NH_3_ also faces several challenges. First, achieving precise control and optimization of vacancy and doping defects in catalysts requires a deep understanding of their synthesis mechanisms and reaction mechanisms, as well as strict control of experimental conditions. Second, ensuring the stability and durability of the catalysts under long-term and high-load operation is crucial for their promotion and widespread use in industrial applications. To overcome these challenges, researchers can employ interface engineering techniques, such as surface modification, coating, or the design of interfacial binding layers, to enhance the surface energy and reactivity of the materials, effectively inhibit vacancy diffusion and aggregation, and improve the stability of the vacancy structure. Moreover, combining advanced characterization techniques and theoretical calculations to investigate the impact mechanisms of vacancy and doping defects on catalyst performance will provide strong support for designing more efficient and stable NO reduction catalysts. In the process of converting NO to NH_3_, vacancy and dopant defect materials exhibit significant research and application potential. However, these materials face a series of challenges in practical industrial applications. Firstly, precise control and optimization of vacancies and dopant defects in the catalyst is a complex task. Secondly, the stability and durability of the catalyst under long-term high-load operation are crucial for its industrial adoption and widespread use. Lastly, the feasibility and economic viability of scaling up the production of defect-engineered catalysts are also significant challenges. Developing cost-effective raw materials and production methods, optimizing preparation processes, and establishing standardized quality control measures are essential for achieving industrial-scale production. By comprehensively addressing these challenges and employing innovative solutions along with interdisciplinary research collaboration, defect-engineered catalysts can be further advanced in industrial applications. This will not only play a key role in the conversion of NO to NH_3_ but also expand their application to other catalytic processes, providing new solutions for a broader range of industrial fields.
